# Transthyretin amyloid deposition in ligamentum flavum (LF) is significantly correlated with LF and epidural fat hypertrophy in patients with lumbar spinal stenosis

**DOI:** 10.1038/s41598-023-47282-7

**Published:** 2023-11-16

**Authors:** Kazuya Maeda, Kazuki Sugimoto, Masayoshi Tasaki, Takuya Taniwaki, Takahiro Arima, Yuto Shibata, Makoto Tateyama, Tatsuki Karasugi, Takanao Sueyoshi, Tetsuro Masuda, Yusuke Uehara, Takuya Tokunaga, Satoshi Hisanaga, Masaki Yugami, Ryuji Yonemitsu, Katsumasa Ideo, Kozo Matsushita, Yuko Fukuma, Masaru Uragami, Junki Kawakami, Naoto Yoshimura, Kosei Takata, Masaki Shimada, Shuntaro Tanimura, Hideto Matsunaga, Yuki Kai, Shu Takata, Ryuta Kubo, Rui Tajiri, Fuka Homma, Xiao Tian, Mitsuharu Ueda, Takayuki Nakamura, Takeshi Miyamoto

**Affiliations:** 1 Department of Orthopedic Surgery, Faculty of Life Sciences, Kumamoto University, 1-1-1 Honjo, Chuo-ku, Kumamoto, 860-8556 Japan; 2 Department of Neurology, Faculty of Life Sciences, Kumamoto University, 1-1-1 Honjo, Chuo-ku, Kumamoto, 860-8556 Japan; 3 Department of Oral and Maxillofacial Surgery, Faculty of Life Sciences, Kumamoto University, 1-1-1 Honjo, Chuo-ku, Kumamoto, 860-8556 Japan; 4 Department of Dentistry and Oral Surgery, Keio University School of Medicine, 35 Shinano-Machi, Shinjuku-ku, Tokyo, 160-8582 Japan

**Keywords:** Medical research, Spinal cord diseases

## Abstract

Lumbar spinal stenosis (LSS) is a degenerative disease characterized by intermittent claudication and numbness in the lower extremities. These symptoms are caused by the compression of nerve tissue in the lumbar spinal canal. Ligamentum flavum (LF) hypertrophy and spinal epidural lipomatosis in the spinal canal are known to contribute to stenosis of the spinal canal: however, detailed mechanisms underlying LSS are still not fully understood. Here, we show that surgically harvested LFs from LSS patients exhibited significantly increased thickness when transthyretin (TTR), the protein responsible for amyloidosis, was deposited in LFs, compared to those without TTR deposition. Multiple regression analysis, which considered age and BMI, revealed a significant association between LF hypertrophy and TTR deposition in LFs. Moreover, TTR deposition in LF was also significantly correlated with epidural fat (EF) thickness based on multiple regression analyses. Mesenchymal cell differentiation into adipocytes was significantly stimulated by TTR in vitro. These results suggest that TTR deposition in LFs is significantly associated with increased LF hypertrophy and EF thickness, and that TTR promotes adipogenesis of mesenchymal cells. Therapeutic agents to prevent TTR deposition in tissues are currently available or under development, and targeting TTR could be a potential therapeutic approach to inhibit LSS development and progression.

## Introduction

Lumbar spinal stenosis (LSS) is a degenerative disease that causes back pain, numbness in the lower extremities, and intermittent claudication due to compression of nerve tissue in the lumbar spinal canal. Incidence increases with age: it is seen in up to 47% of those aged 60 years and older, and approximately 600,000 people in the U.S. undergo surgery for LSS each year^1,2^. Primary causes of LSS are disc degeneration, intervertebral joint hypertrophy, and ligamentum flavum (LF) hypertrophy^3–5^. LF hypertrophy is reportedly promoted by TGFβ1, Angptl2, MMP-2, and IL-6, among other factors^3,6–9^. However, mechanisms underlying LF hypertrophy remain unclear.

The protein transthyretin (TTR), which is responsible for amyloidosis, is reportedly involved in LF hypertrophy^10–12^. Amyloidosis is an intractable disease characterized by deposition of extracellular insoluble amyloid derived from several proteins^13^. Thirty-seven proteins have been reported as amyloid sources^14^. The physiological function of TTR protein, which is produced primarily in the liver, is to bind thyroid hormones and retinol and transport those factors through the blood^14^. However, TTR can be deposited in various organs including ligaments^15^, and amyloidosis due to TTR deposition has been reported in patients with carpal tunnel syndrome and cardiac amyloidosis^16–20^.

Spinal epidural lipomatosis (SEL) is a rare disease that promotes various symptoms similar to LSS. SEL is marked by excessive growth of fatty tissue in the spinal epidural space, and the resultant increase in epidural fat (EF) compresses the spinal cord, causing back pain and numbness in lower extremities, symptoms that can be improved to some extent by weight loss or surgical decompression^21,22^. Chronic exposure to endogenous or exogenous steroids^23–26^, obesity^27–29^, and hypertension^29^ are reported risk factors for SEL. However, mechanisms underlying the increase in epidural fatty tissues in the spinal canal are unknown.

This study shows that LF exhibiting TTR deposition was significantly thicker than LF lacking TTR. We demonstrate that LF hypertrophy is associated with TTR deposition based on multiple regression analysis. We also show that EF was significantly thicker in patients with TTR-positive compared to those with TTR-negative LF. Spinal canal stenosis due to SEL was indicated based on an increased EF/SpiC ratio. Finally, we show that TTR deposition is significantly associated with an increased EF/SpiC ratio based on multiple regression analysis.

## Materials and methods

### Patients

This study was conducted after approval was obtained from the Kumamoto University Ethics Committee and was performed by relevant guidelines/regulations. Written informed consent was received from each patient. LF and EF samples used in this study were provided by LSS patients who underwent surgery for lumbar spinal canal stenosis at Kumamoto University Hospital from June 2020 to November 2022.

### Congo red staining

Harvested LF and EF tissues were fixed in 4% paraformaldehyde (PFA), embedded in paraffin, and cut into 4 μm thick sections. After stained with phenol Congo red, sections were examined under polarized light for the presence of green birefringence.

Staining with 1-fluoro-2,5-bis[(*E*)-3-carboxy-4-hydroxystyryl]benzene (FSB).

Harvested LF and EF tissues were fixed in 4% paraformaldehyde (PFA), embedded in paraffin, and cut into 4 μm thick sections. After staining 1 h in a working solution (0.01% vol/vol in PBS) of FSB (F308, Dojindo, Kumamoto, Japan), sections were observed under UV light (V excitation) (excitation, 405 nm; emission, 420–520 nm)^30^.

### Immunohistochemistry

Deparaffinized sections were incubated 15 min in a 5 mM periodic acid solution to block endogenous peroxidases. Protein Block (ab64226, Abcam, Cambridge, UK) was used to block nonspecific background staining. Polyclonal rabbit anti-human prealbumin (A0002, Dako/Agilent Technologies, Santa Clara, CA) and monoclonal rabbit anti-human apolipoprotein A1 (ab52945, Abcam, Cambridge, UK) antibodies were used at 1:100 dilution as primary antibodies, and a polyclonal goat anti-rabbit immunoglobulin/HRP antibody (P0448, Dako/Agilent Technologies) was used at 1:100 dilution as a secondary antibody. Reactivity was visualized using DAB (D006, Dojindo, Kumamoto, Japan), and sections were counterstained with hematoxylin.

### Measurement of LF and EF thickness

LF thickness at the facet joint of the affected spinal level was measured using axial T2-weighted magnetic resonance images, which were acquired before surgery. Measuring tools within the MRI viewer were used for evaluation. Bilateral measurements were made, and the arithmetic mean of the two values was recorded. The anteroposterior diameters of both the EF and the spinal canal (SpiC) at the facet joint at the affected spinal level were measured on axial T2-weighted magnetic resonance images, and then the EF/SpiC index (ratio of the anteroposterior diameter of EF to the anteroposterior diameter of the SpiC) was calculated as an indicator of EF thickness, as described by Fujita et al.^27^. LF thickness and the EF/Spic index were measured three times, and average measurements served as the final values. Investigators were blinded to TTR status during data collection.

### ADSC culture and induction of adipocyte differentiation

Human Adipose-Derived mesenchymal Stem Cells (ADSCs) were purchased from Lonza (PT-5006, Basel, Switzerland), seeded in 6-well plates filled with medium from the ADSC-BulletKit™ (PT-4505, Lonza), and cultured at 37 °C in a 5% CO_2_ humidified incubator. The culture medium was changed twice a week. ReagentPack™ Subculture Reagents (CC-5034, Lonza) were used for ADSC passages. Subsequent experiments were performed using cells from primary cultures up to the third passage. Adipocyte differentiation induction medium supplied with the PGM™-2 preadipocyte growth medium-2 BulletKit™ (PT-8002, Lonza) was used for adipocyte differentiation of ADSCs, according to the manufacturer's instructions. As a control, we used the maintenance medium supplied with the kit.

To analyze TTR effects on adipocyte differentiation of ADSCs, ADSCs were seeded in 96-well plates and after full confluence, adipocyte differentiation was induced using adipocyte differentiation induction medium (AM) with or without 5 μM TTR. As a control, cultures were performed in a maintenance medium (MM) with or without 5 μM TTR. TTR was synthesized and used as previously described^31^.

### Oil red O staining

Adipocytes derived from ADSCs were washed three times with phosphate-buffered saline (pH 7.4) and fixed with 4% paraformaldehyde phosphate buffer solution for 1 h at room temperature. Adipocytes were washed three times with distilled water and stained with oil-red O staining solution for 20 min at room temperature with gentle agitation. The staining solution was made by dissolving 30 mg of oil red O (#154-02072, Fujifilm Wako Pure Chemical Corp, Osaka, Japan) in 10 ml of 100% isopropanol, stirring, mixing with distilled water at a 3:2 ratio, and filtering. After staining, the excess stain was removed from adipocytes with 60% isopropanol, and cells were washed twice with distilled water before being photographed under a light microscope. Accumulated lipids were extracted in 200 μl 100% isopropanol and measured by reading extract absorbance at OD 490 nm using a microplate reader (1681135JA, Bio-rad, Hercules, CA).

### Real-time polymerase chain reaction analysis

RNA was extracted using TRIzol (Invitrogen, Life Technologies, Carlsbad, CA). RNA was then reverse-transcribed using PrimeScript™ RT Master Mix (Perfect Real Time) (RR036A, Takara Bio, Ozu, Japan), followed by real-time polymerase chain reaction (PCR) using a Thermal Cycler Dice® Real Time System III (TP950, Takara Bio) and TB Green® Premix Ex Taq™ II (Tli RNaseH Plus) (RR820S, Takara Bio). The relative abundance of target transcripts was normalized to the expression of *GAPDH mRNA*. The following primers were used in the study: *GAPDH*-forward, 5′-CCACCCATGGCAAATTCCATGGCA-3′; *GAPDH*-reverse, 5′-TCTAGACGGCAGGTCAGGTCCACC-3′; *PPARγ*-forward, 5′-GCCAAGCTGCTCCAGAAAAT-3′; and *PPARγ*-reverse, 5′-TGATCACCTGCAGTAGCTGCA-3′.

### Statistical analysis

Data were analyzed using EZR on R commander version 1.54^32^. The following statistical tests were performed: t-test to compare two groups of continuous variables, Pearson’s correlation test to assess the linear relationship between two continuous variables, multiple regression analysis to predict a dependent variable from several independent variables, and one-way ANOVA to compare more than two groups of continuous variables. The level of significance was set at 0.05 for all tests.

## Results

### Amyloid deposition and TTR-positivity of specimens from patients with LSS

**Figure 1:**
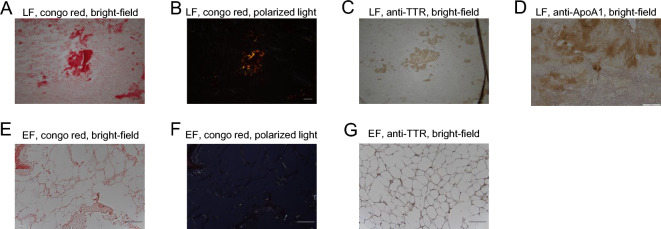
Amyloid deposits in the LF and EF in patients with lumbar spinal canal stenosis. The ligamentum flavum (LF) (**A**, **B**, **C** and **D**) and epidural fat (EF) (**E**, **F** and **G**) were removed from patients with lumbar canal stenosis and stained with Congo red to detect amyloid deposition (**A**, **B**, **E** and **F**) or with anti-human transthyretin (TTR) antibody (**C** and **G**) or with anti-human anti-Apolipoprotein AI(ApoA1) antibody (**D**) and observed under a microscope in bright-field (**A**, **C**, **D**, **E**, and **G**) or under polarized light (**B** and **F**). Scale bars, 100 μm.

The study included 87 patients with confirmed LSS based on MRI and symptoms who had undergone surgery (Table 1). After LF samples were surgically removed from patients, Congo red staining was performed to detect amyloid deposition, and we detected positive amyloid deposition in 67 of 87 LF specimens (77.0%) (Fig. 1A,B). Amyloid deposition in Congo red-positive LFs was confirmed was confirmed based on overlapping FSB positivity (Supplementary Fig. 1). Immunostaining using anti-TTR antibodies showed that 47 of 87 LF cases (54.0%) were TTR-positive (Table 1, Fig. 1C). Among 40 TTR-negative samples, 20 of 40 were Congo red-positive, and 10 of those (50.0%) were positively stained with anti-Apolipoprotein AI (ApoA1) antibody (Fig. 1D). Similarly, all 6 EF specimens showed amyloid deposition (Fig. 1E,F), and 1 case was TTR- positive (Fig. 1G). None of the remaining 5 TTR-negative EF cases was positively stained with anti-ApoA1 antibody.

**Table 1:** Characteristics of LSS patients with or without TTR + deposits in ligamentum flavum (LF).

Characteristic		Transthyretin+	Transthyretin−	*P*-value
Frequency of TTR positivity	n (%)	47 (54.0%)	40 (46.0%)	
Males/females	n	36/11	26/14	0.234
Age	years	76.83 ± 9.38	72.75 ± 11.89	0.077
Body Mass Index	kg/m^2^	25.09 ± 3.55	23.82 ± 3.85	0.112
Steroid use (yes/no)	n	2/45	6/34	0.084
Diabetes mellitus (yes/no)	n	5/42	7/33	0.355
Mean LF thickness	mm	4.89 ± 0.97	4.07 ± 1.16	0.0005
EF/SpiC index		0.44 ± 0.16	0.35 ± 0.15	0.005
Level of harvested LF	n			
L1/2		3	0	
L2/3		7	6	
L3/4		17	10	
L4/5		19	18	
L5/S		1	6

The mean age at the time of surgery did not differ statistically between LSS patients with (TTR +) or without (TTR-) TTR deposits in LF (Table 1). Similarly, parameters including body mass index (BMI), gender ratio, steroid use, or diabetes incidence at the time of surgery did not differ statistically between TTR + and TTR- patients (Table 1). TTR + /ApoA1- specimens were thicker than TTR-/ApoA1 + specimens (4.89 mm versus 4.11 mm, *p* = 0.029, Supplementary Table 1). The thickness of TTR-/ApoA1 + LFs was comparable to that of Congo red- LFs (4.11 mm versus 4.00 mm, *p* = 0.801, Supplementary Table 1).

### TTR deposition is significantly associated with either LF thickness or the EF/SpiC ratio

**Figure 2:**
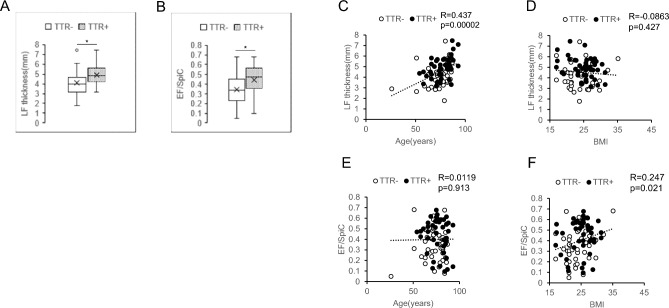
LF and EF from TTR-positive LFs are significantly thicker than those from TTR-negative LFs. LF and EF thickness was determined by MRI imaging, and EF thickness was calculated as the ratio (EF/SpiC) of the anteroposterior diameter of EF to the anteroposterior diameter of the spinal canal (SpiC) and compared in TTR + and TTR- patients (**A** and **B**). Relationships between LF thickness and BMI (**C**), LF thickness and BMI (**D**), the EF/SpiC ratio and age at surgery (**E**), and the EF/SpiC ratio and BMI (**F**) were analyzed. Correlation coefficients (R) and p values (p) are shown.

Next, LF thickness in each patient was analyzed by MRI data obtained before surgery. LFs from patients whose specimens were TTR + were significantly thicker than those from TTR- specimens (4.89 mm versus 4.07 mm, *p* = 0.0005, Table 1). EF thickness in the spinal canal, evaluated by MRI, was significantly greater in TTR + than in TTR- specimens based on analysis of respective EF/SpiC ratios (0.44 versus 0.35, *p* = 0.005) (Fig. 2A,B). There was a significant positive correlation between LF thickness and age (Fig. 2C) and between EF/SpiC ratio and BMI (Fig. 2F). However, there was no significant correlation between LF thickness and BMI or EF/SpiC ratio and age (Fig. 2D,E).

Multiple regression analysis was performed with LF thickness and EF/SpiC ratio as objective variables, while age, the presence of TTR deposition in LF, and BMI were analyzed as explanatory variables. Age (β = 0.367, *p* = 0.0003) and the presence of TTR deposition (β = 0.301, *p* = 0.002) were significantly associated with LF thickness (Table 2), while TTR deposition in LF was significantly associated with the EF/SpiC ratio (β = 0.262, *p* = 0.017) (Table 3).

**Table 2:** Analysis of LF thickness: multiple regression model.

Variables	Coefficient (B)	Standardized coefficient (β)	SE	t	p	95%CI
Age	0.039	0.367	0.010	3.702	0.0003	0.018–0.060
TTR	0.682	0.301	0.221	3.087	0.002	0.243–1.121
BMI	− 0.015	− 0.050	0.030	− 0.506	0.614	− 0.075–0.045

In stepwise regression. Adjusted R^2^ = 0.248.

**Table 3:** Analysis of EF/SpiC: multiple regression model.

Variables	Coefficient (B)	Standardized coefficient (β)	SE	t	p	95%CI
Age	0.0002	0.011	0.002	0.103	0.918	− 0.003–0.003
TTR	0.085	0.262	0.035	2.440	0.017	0.016–0.155
BMI	0.010	0.205	0.005	1.891	0.062	− 0.0005–0.018

In stepwise regression. Adjusted R^2^ = 0.097.

### Adipocyte differentiation of mesenchymal cells is enhanced by TTR

**Figure 3:**
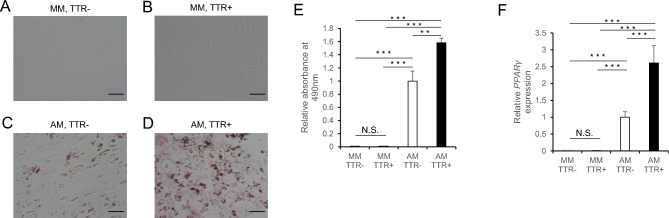
Adipocyte differentiation of human ADSCs is enhanced by recombinant transthyretin (TTR). ADSCs were cultured 13 days in control maintenance medium (MM) (**A** and **B**) or adipocyte differentiation induction medium (AM) (**C** and **D**), either with (**B** and **D**) or without (**A** and **C**) recombinant transthyretin (TTR). Cells were then stained with oil Red O and observed under a microscope (**A**–**D**). Scale bar, 100 μm. (**E**) ADSCs were cultured 13 days in MM or AM with or without TTR, stained with oil Red O, dissolved in isopropanol, and extract absorbance at 490 nm was determined to quantify adipogenic differentiation. (**F**) ADSCs were cultured 96 h in MM or AM with or without TTR, and *PPARγ* expression was analyzed by real-time PCR. Data represent mean relative absorbance ± SD (**E**) or *PPARγ* expression relative to *GAPDH* ± SD (**F**) (N.S., not significant; **p < 0.01; ***p < 0.001).

Since TTR deposition in LF was significantly associated with the EF/SpiC ratio, we assessed the role of TTR on adipocyte differentiation (Fig. 3). To do so, we cultured mesenchymal cells (ADSCs) in adipocyte differentiation induction medium in the presence or absence of TTR for 13 days, and then stained cells with oil red O to evaluate adipocyte differentiation. ADSCs cultured in differentiation induction medium plus TTR were more intensely stained by oil red O than those cultured without TTR (Fig. 3C,D). ADSCs cultured in maintenance medium were not stained by oil red O, even in the presence of TTR (Fig. 3A,B), suggesting that TTR stimulates adipocyte differentiation induced by factors in the medium. Adipocyte differentiation of ADSCs in differentiation induction medium, as quantified by measuring oil red O absorbance, was significantly stimulated by TTR (*p* = 0.002) (Fig. 3E).

We next asked how TTR stimulates adipocyte differentiation by ADSCs. After 13 days of incubation in adipocyte induction medium containing recombinant TTR, ADSCs were Congo red-positive, and deposits showed yellow-green birefringence under polarized light (Supplementary Fig. 2A and B), suggesting that TTR in the culture medium had been converted to amyloid fibril on the cell surface and had then transduced adipocyte differentiation signals. We then extracted RNA from ADSCs cultured for 96 h in either differentiation or maintenance medium and analyzed expression of *PPARγ*, the master transcription factor for adipocyte differentiation, by realtime PCR. *PPARγ* expression was comparable in ADSCs cultured in maintenance medium with or without TTR (Fig. 3C and D). By contrast, *PPARγ* expression was significantly upregulated in ADSCs cultured in adipocyte differentiation induction medium, and that expression was higher in the presence of TTR (*p* < 0.0001) (Fig. 3F).

Finally to determine whether pre-incubation with TTR altered induction capacity of cell culture medium, we added TTR to adipocyte induction medium and allowed it to incubate 3 days at 37 °C before adding it to ADSC cells, which were cultured for 13 more days in that media (Supplementary Fig. 3), since TTR was reportedly had proteolytic activity^33^. Based on analysis of both oil red O absorbance and *PPARγ* expression (Supplementary Fig. 3A and B), ADSCs grown in TTR-preincubated media did not exhibit adipocyte differentiation, suggesting that pre-incubation of media with TTR degrades a factor required for differentiation.

## Discussion

LF hypertrophy and EF thickening are both causes of LSS, but mechanisms underlying those changes have not been fully characterized in patients. In the present study, we found that 67 of 87 LFs (77.0%) were positive for amyloid and 47 (54.0%) were also positive for TTR, supporting findings of others who reported a high frequency of TTR deposition in LFs from patients with LSS^11,34^. We also detected Apolipoprotein AI amyloid in half of the TTR-negative/Congo red-positive samples, findings consistent with a previous study^35^. We show that TTR deposition in LFs surgically removed from LSS patients was significantly correlated with LF and EF thickening. TTR deposition in LF of LSS patients has been reported to promote LF thickening^10–12^. However, here we show that TTR deposition in LF is significantly associated with both LF hypertrophy and EF thickening, both of which promote LSS.

Various factors are thought to promote LF hypertrophy, including elevated expression of Matrix metalloproteinase 13 (MMP13) and sorbitol accumulation in LF^36,37^; however, mechanisms underlying this process remain unknown. Among potentially relevant factors, aging is considered a major cause of LF hypertrophy^3^. Here, we observed a significant positive correlation between LF thickness and age. However, our multiple regression analysis revealed that TTR is associated with LF hypertrophy^37^, and although diabetes is a known risk for SEL, diabetes incidence did not differ between our TTR-positive and TTR-negative groups. Obesity and steroid use have also been identified as risks for SEL^27–29^, and indeed we observed a positive correlation between BMI and EF thickness in our patients. However, we observed no statistically significant difference in BMI and steroid use between TTR-positive and TTR-negative groups. Interestingly, although we observed a positive correlation between BMI and EF thickness, the presence of TTR deposition in LF was significantly related to EF thickness, independently of BMI, based on multiple regression analyses. These findings suggest that TTR deposition in LF is likely an independent cause of LF hypertrophy and SEL. Other amyloid-related diseases, such as wild-type transthyretin amyloidosis (ATTRwt), are more common in older males^38–40^. In this study, there was no significant difference in the age and ratio of males and females in both TTR-positive and TTR-negative groups.

Amyloidosis is a disease caused by amyloid deposition in various tissues. When amyloid deposition occurs in nerve cells, it causes various neurodegenerative conditions such as Alzheimer's or Parkinson's diseases^41,42^. Moreover, amyloid deposition in the heart causes amyloid cardiomyopathy, which includes hereditary transthyretin amyloidosis and immunoglobulin light chain amyloidosis^14,43^. In the musculoskeletal system, amyloid deposition is a reported cause of carpal tunnel syndrome^18,20^, and bilateral carpal tunnel syndrome is considered a predictor of cardiac amyloidosis since it often develops before cardiac amyloidosis^18^. Approximately 8% of patients with wild-type transthyretin amyloidosis (ATTRwt) are also reportedly treated for LSS, and the presence of ligament disorders including LSS may be a predictor of hospitalization and worsening heart failure^44^. Screening for TTR-induced cardiomyopathy (ATTR-CM) in LSS patients has also been undertaken, but no association between ATTR-CM and LSS is reported^34^. Thus, future longitudinal studies are needed to determine whether LSS patients with TTR-positive LFs subsequently develop systemic ATTR disease. Amyloid is also frequently deposited in fatty tissues, and biopsy of subcutaneous abdominal tissue is a diagnostic that can be useful in determining the type of amyloidosis^45^. Nonetheless, the effects of amyloid deposition on fat are unclear, and further studies are required to address this question. Here, however, we show that EF is also a target of amyloid deposition.

The present study has some limitations. Firstly, mechanisms by which amyloid deposition in tissues promotes disease development are unclear. Secondly, while we demonstrate that TTR stimulates adipocyte differentiation of ADSCs in vitro and expression of *PPARγ*, which is required for their differentiation, it is unclear how TTR promotes adipogenesis and why only 1 of 6 EFs was TTR-positive. TTR monomers reportedly form oligomers^31^, and indeed, we show that TTR in the culture medium is converted to amyloid fibrils on the cell surface and transduces adipocyte differentiation signals. Since 5 TTR-negative EFs were ApoA1-negative, future studies are needed to identify amyloids deposited on those EFs. Thirdly, since the study only included patients with lumbar spinal stenosis, we do not know whether our findings can be generalized to patients with other diseases or to healthy individuals. Further studies are needed to define mechanisms underlying LF and EF hypertrophy and how adipocyte differentiation is stimulated by amyloid deposition.

Overall, however, our study revealed a significant correlation between TTR deposition and LF and EF thickening, suggesting that TTR deposition likely underlies LF hypertrophy and SEL in LSS patients. TTR forms a homo-tetrameric protein in human plasma, but dissociation of that tetramer and misfolding of TTR monomers can result in the formation of amyloid fibrils and subsequent deposition in tissues^14^. Currently, a reagent that binds the TTR tetramer, inhibits its dissociation and stabilizes the tetramer is available clinically and has been shown to reduce all-cause mortality in TTR amyloid cardiomyopathy^46^. Therefore, inhibiting TTR deposition in LFs and EFs may be a potential therapeutic strategy for preventing LSS progression.

### Supplementary Information

Supplementary Information.

## Data Availability

The datasets generated during and/or analyzed during the current study are available from the corresponding author upon reasonable request.
