# Supplementary material for: Transthyretin amyloid deposition in ligamentum flavum (LF) is significantly correlated with LF and epidural fat hypertrophy in patients with lumbar spinal stenosis
Source: Sci Rep. 2023 Nov 16;13:20019. doi: 10.1038/s41598-023-47282-7 (PMC10654520; doi:10.1038/s41598-023-47282-7)
Supplement: Supplementary file 1 — Supplementary Information. [file 41598_2023_47282_MOESM1_ESM.pdf]

Supplementary Figure Legends

Supplementary figure 1. Comparison of Congo Red and FSB staining.

The ligamentum flavum (LF) was removed from patients with lumbar canal stenosis, and then tissues were sectioned and stained with Congo red to detect amyloid deposition (A) or with FSB (B). Samples were observed under a microscope in bright-field (A) or UV light (B). Scale bars, 100 μm.

Supplementary figure 2. Congo red staining of ADSCs cultured 13 days in adipocyte differentiation induction medium plus TTR.

ADSCs were cultured 13 days in adipocyte differentiation induction medium containing recombinant transthyretin (TTR) and then stained with Congo red and observed under a microscope in bright-field (A) or under polarized light (B). In (B), higher magnification image of area in the white box is shown at lower left corner. Scale bar, 100 μm.

Supplementary figure 3. Adipocyte differentiation induction medium pre-incubated with TTR does not stimulate ADSC differentiation.

Adipocyte induction medium was incubated 3 days with TTR. ADSCs were then cultured 13 days in that media (pre-exposed TTR). Adipocyte induction medium was also incubated 3 days without TTRs, and then added to the ADSC culture with (TTR+) or without (TTR-) TTR. (A) Cells were then stained with Oil Red O, treated with isopropanol, and extract absorbance at 490 nm was determined to quantify adipogenic differentiation. (B) ADSCs were cultured as described above and then analyzed for PPARγ expression based on real-time PCR. Data represent either mean relative absorbance ± SD (A) or PPARγ expression relative to GAPDH ± SD (B).

Supplementary Table 1. Characteristics of LSS patients with various amyloid deposits in LF.

Characteristic		TTR	ApoA1	Unknown	Negative	P-value
Number of samples	n(%)	47(54.0%)	10(11.5%)	10(11.5%)	20(23.0%)	
Males/females	n	36/11	5/5	7/3	14/6	0.421
Age	years	76.83±9.38	75.10±8.52	71.10±12.85	72.40±13.15	0.280
Body Mass Index	kg/m^2	25.09±3.55	23.08±3.93	24.30±4.73	23.95±3.48	0.380
Mean LF thickness	mm	4.89±0.97	4.11±1.18	4.18±1.46	4.00±1.03	0.007
EF/SpiC index		0.44±0.16	0.37±0.17	0.43±0.17	0.29±0.12	0.003

(TTR, Patients with LSS with Transthyretin deposition in the LF; ApoA1, Patients with LSS with Apolipoprotein A1 deposition in the LF; Unknown, Patients with LSS with unidentified amyloid deposition in the LF; Negative, Patients with LSS without amyloid deposition in the LF)

A LF, congo red, bright-field

B LF, FSB, bright-field

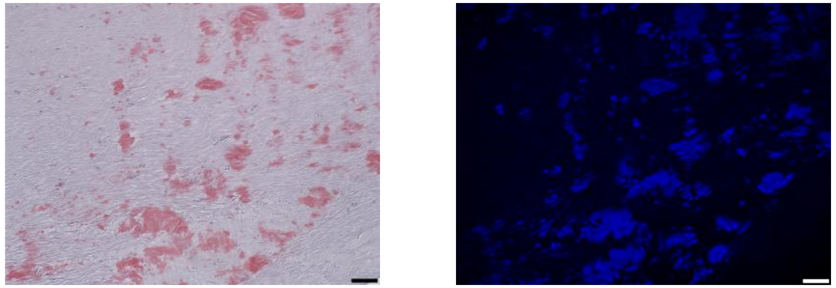

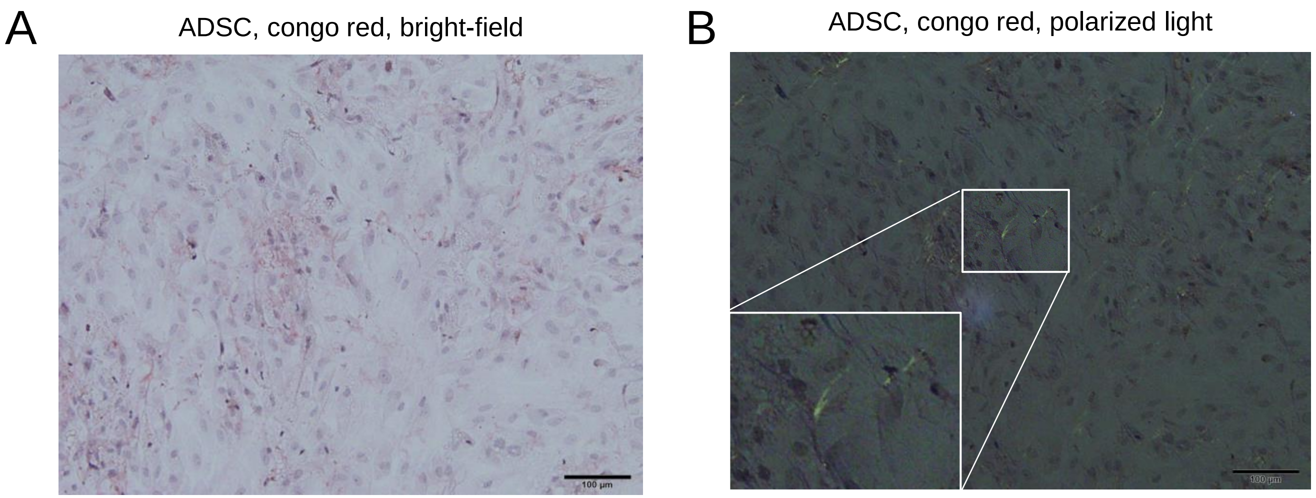
A
ADSC, congo red, bright-field
100 µm
B
ADSC, congo red, polarized light
100 µm

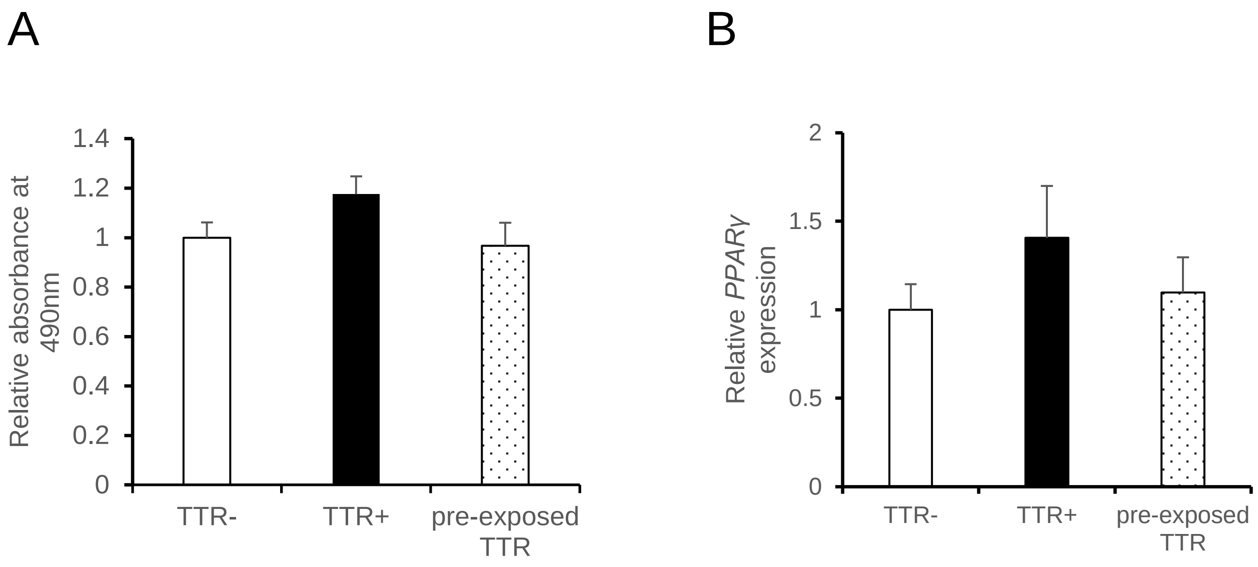
A
Relative absorbance at 490nm
1.4
1.2
1
0.8
0.6
0.4
0.2
0
TTR-
TTR+
pre-exposed TTR
B
Relative PPARγ expression
2
1.5
1
0.5
0
TTR-
TTR+
pre-exposed TTR
